# Age- and gender-specific trends in respiratory outpatient visits and diagnoses at a tertiary pediatric hospital in China: a 10-year retrospective study

**DOI:** 10.1186/s12887-020-2001-x

**Published:** 2020-03-12

**Authors:** Peng Shi, Xiaobo Zhang, Lijuan Liu, Liangfeng Tang, Jing Li, Libo Wang, Albert M. Li, Yang Cao, Hong Xu, Guoying Huang

**Affiliations:** 1 Department of Data Management and Statistics, Children’s Hospital of Fudan University, Shanghai, China; 2 Department of Respiratory Disease, Children’s Hospital of Fudan University, National Children’s Medical Center, Shanghai, 201102 China; 3 Department of Urology, Children’s Hospital of Fudan University, Shanghai, China; 4 Big Data Product Department, Wonders Information Co. Ltd., Shanghai, China; 5 Department of Paediatrics, The Chinese University of Hong Kong, Hong Kong, China; 6 Clinical Epidemiology and Biostatistics, School of Medical Sciences, Örebro University, Örebro, Sweden; 7 Department of Nephrology and Rheumatology, Children’s Hospital of Fudan University, Shanghai, China; 8 Pediatric Heart Center, Children’s Hospital of Fudan University, Shanghai, China

**Keywords:** Respiratory infections, Asthma, Pediatrics, Outpatients, Trend study

## Abstract

**Background:**

Respiratory infections are one of three leading causes of childhood mortality, and worldwide increase and recent plateau in childhood asthma has been reported. However, data on trends of respiratory diseases over long period of time is limited. This study aimed to determine the trends of respiratory disease outpatient visits (ROVs) and diagnoses (RODs) in one of the largest children’s teaching hospitals in China between 2009 and 2018.

**Methods:**

A retrospective study based on routine administrative data was designed and implemented according to the RECORD statement. Demographic details and diagnoses of the outpatients < 18 years visiting the respiratory department of the hospital were extracted from the Hospital Information System. Age- and gender-specific trends were illustrated by calculating average annual growth rate (AAGR) for ROVs and comparing change of proportion for different RODs over time.

**Results:**

There were 698,054 ROVs from 285,574 children (40.4% female). AAGR of ROVs was 15.2%. Children aged 4 to < 7 years had a faster increase than other age groups. Bronchitis (27.6%), pneumonia (18.5%), pneumonia affecting other systems (18.4%), asthma and status asthmaticus (10.7%), and vasomotor and allergic rhinitis (9.2%) accounted for 84.4% of all RODs. The proportion of bronchitis decreased across years, with the concomitant increasing trend in the proportion of pneumonia. Age-specific trend in diagnoses showed greater proportion of asthma in all visits for the children aged 7 to < 18 years than younger children. Gender-specific trend in diagnoses showed the proportion of asthma was greater for males but the AAGR was greater for females.

**Conclusion:**

The persistent upward trend in ROVs was observed among children at different ages and a gender difference was also seen. In contrast to what has been reported, burden of asthma and allergies diseases continues to increase locally.

## Background

Respiratory infections are still one of three leading causes of child mortality worldwide [1–3]. Each year childhood respiratory infections bring along a significant burden to our healthcare system in terms of manpower and resource utilization [4–6]. The National Surveillance for Asthma in the United State (US) from 1980 to 2013 reported the increasing incidence of respiratory infections and prevalence of asthma [7–9]. Compared with the report from the US, studies in Shanghai also showed an almost five-fold increased prevalence of asthma among children in China, from 2.1% in 1990 to 10.2% in 2011, with a higher prevalence of asthma in boys than in girls in the age group of 3–7 years [10]. These studies suggested age and gender differential phenomena on the increasing asthma prevalence. However, the full picture of respiratory outpatient visits (ROVs) and diagnoses (RODs) over a longer term period, especially the age- and gender-specific trends, has not been well documented, partly due to the lack of computerized databases containing diagnosis-specific outpatient data. In this study, we used 10-year routine administrative data and reported age- and gender-specific trends in ROVs and RODs among children visiting one of the largest tertiary-level pediatric hospitals in China.

## Methods

### Study design

This was a hospital-based retrospective study using routine administrative data and was implemented according to the RECORD statement [11]. The primary objective was to describe age- and gender-specific trends in ROVs and RODs over a 10-year period. Ethics approval was obtained from the Children’s Hospital of Fudan University Ethics Committee (No. 2019–188).

### Participants

We included visits for patients aged < 18 years with any outpatient visit to the respiratory department of the hospital. The registration number for the visit (Visit ID) was the identification code for every visit of patient, and the patient number (Patient ID) was another identification code for different patients. We selected participants using the criteria: (1) the registration date between Jan 1st, 2009 and Dec 31st, 2018, (2) the registration department was respiratory department, and (3) patients aged < 18 years. We validated the data of participants in each year using the annual reports of the hospital.

### Setting

The setting of this study was the Children’s Hospital of Fudan University, a tertiary, teaching hospital and a National Children’s Medical Center located in Shanghai, China.

### Data sources and data linkage

**Fig. 1:**
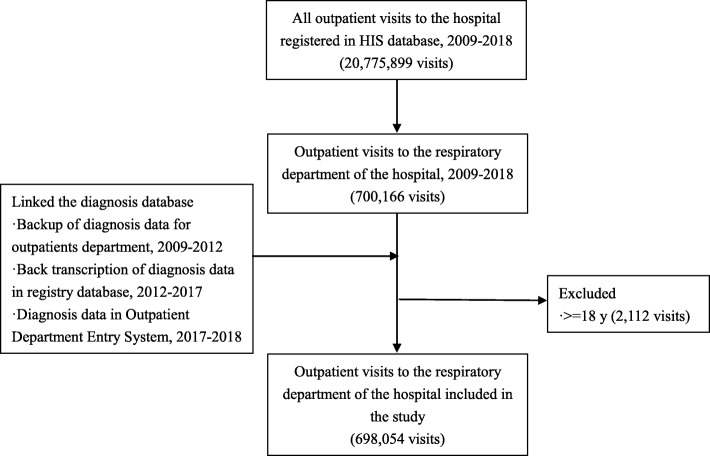
Flowchart illustrating selection of participants and data linkage process

Our data were based on linking hospital outpatient registration data in the health information system (HIS) from 2009 to 2018 to diagnosis records in different databases during the three upgrading phases of the HIS: backup of diagnosis data for outpatient department (2009–2012), back transcription of diagnosis data in registry database (2012–2017), and diagnosis data in Outpatient Department Entry System (2017–2018) for all patients who visited the respiratory department in the hospital. The participant selection and data linkage process was shown in Fig. 1.

### Variable coding and grouping

1. *Outpatient visit* The Visit ID was unique in the HIS for every visit of patient.
2. *Age categories of patient* Age of patient was calculated by the difference between birth date and visit date and classified as < 1 year, 1 to < 4 years, 4 to < 7 years, 7 to < 12 years, and 12 to < 18 years.
3. *Visit diagnoses* Each visit recorded in the HIS system includes outpatients diagnoses, with the first being considered the primary diagnosis. Diagnoses were coded by the International Classification of Diseases, 10th Revision (ICD-10). We selected the primary diagnosis for each visit and categorized the diagnoses based on the category codes (Additional file 1: Appendix 1).

### Data quality

Completeness and distribution were assessed and compared for key variables (age, gender, and diagnosis). Missing rates of key variables were listed in Additional file 1: Appendix 2 and distribution comparison was used to assess the bias due to missing data.

### Statistical methods

We used SQL server 2010 and Python platform for data processing and IBM SPSS Statistics 22.0 for data analysis. For descriptive analyses, number and percentage (%) was used for qualitative variables, median and interquartile range (IQR) or mean ± SD for continuous variables according to its distribution. We analyzed the 10-year trends using two indicators, including the number of ROVs and the proportion of RODs. The trend in ROVs was assessed by average annual growth rate (AAGR) to show the constant ratio through the study period. The AAGR was calculated in terms of a geometric growth expressed as (X_*n*_/X_*0*_)^(1/*n*)^-1 (where X_*0*_ is the value of the baseline and X_*n*_ is the value of the *n*th year)) [12]. A simple linear regression was used to model the increasing trend in ROVs with year. The change in RODs was compared by the proportion of the selected diagnosis to the whole RODs. Chi-squared test for trend was used to test whether there is a systematic increase or decrease in the proportions through the years. Distribution of patients with missing data through the years was compared using the chi-squared test. A two-tailed *P* value of less than 0.05 was considered statistically significant. Sample sizes were large in this study, therefore effect sizes were more meaningful than *P* value in our study for trend analysis and distribution comparisons [13].

## Results

### Patient characteristics

Total of 285,574 patients attended to the respiratory outpatient department of the hospital during the 10-year study period. Preschool children aged < 7 years old accounted for 88.0% of all the children. The number of children aged 1 to < 4 years was twice of those aged < 1 years and 4 to < 7 years. Males accounted for 59.6% of the participants, 39.4% of the patients were covered by government insurance and 60.6% were self-finance. Respiratory specialist clinic provided medical service for 46.5% patients mainly with special respiratory diseases. The doctors with associate professor position or above provided medical service for 52.3% patients in the expert-clinic. The median and the 25th and the 75th percentiles of visits per children were 1 and (1, 3), and the number of visits had no significant difference between the patients covered by government insurance and those who were self-finance [1(1, 3) vs. 1(1, 2)] (Table 1).

**Table 1:** Characteristics of patients, 2009–2018

	Patients visited the respiratory outpatient department
*n* of patients	285,574
Age, *n*(%)	
< 1 year	74,034(23.0)
1 to < 4 years	132,178(41.1)
4 to < 7 years	76,818(23.9)
7 to < 12 years	33,825(10.5)
12 to < 18 years	4512(1.4)
Total^a^	321,368
Sex, *n*(%)	
Male	170,192(59.6)
Female	115,382(40.4)
Payer type, *n*(%)	
Government insurance	112,495(39.4)
Self-finance	173,079(60.6)
Clinic types, *n*(%)	
Specialist Clinic	170,808(46.5)
Expert-clinic	192,081(52.3)
Disease-specific Clinic	4434(1.2)
Total^b^	367,323
Average visits per patients	
Government insurance	
Mean ± SD	2.9 ± 4.0
Median (IQR)	1(1,3)
Self-pay	
Mean ± SD	2.1 ± 2.7
Median (IQR)	1(1,2)
Total	
Mean ± SD	2.4 ± 3.3
Median (IQR)	1(1,3)

^a^A child could contribute data to multiple age groups as he or she visited the department of respiratory disease in different year

^b^A child could contribute data to multiple clinic types as he or she visited the department of respiratory disease in different type

### Overall trend in ROVs

**Fig. 2:**
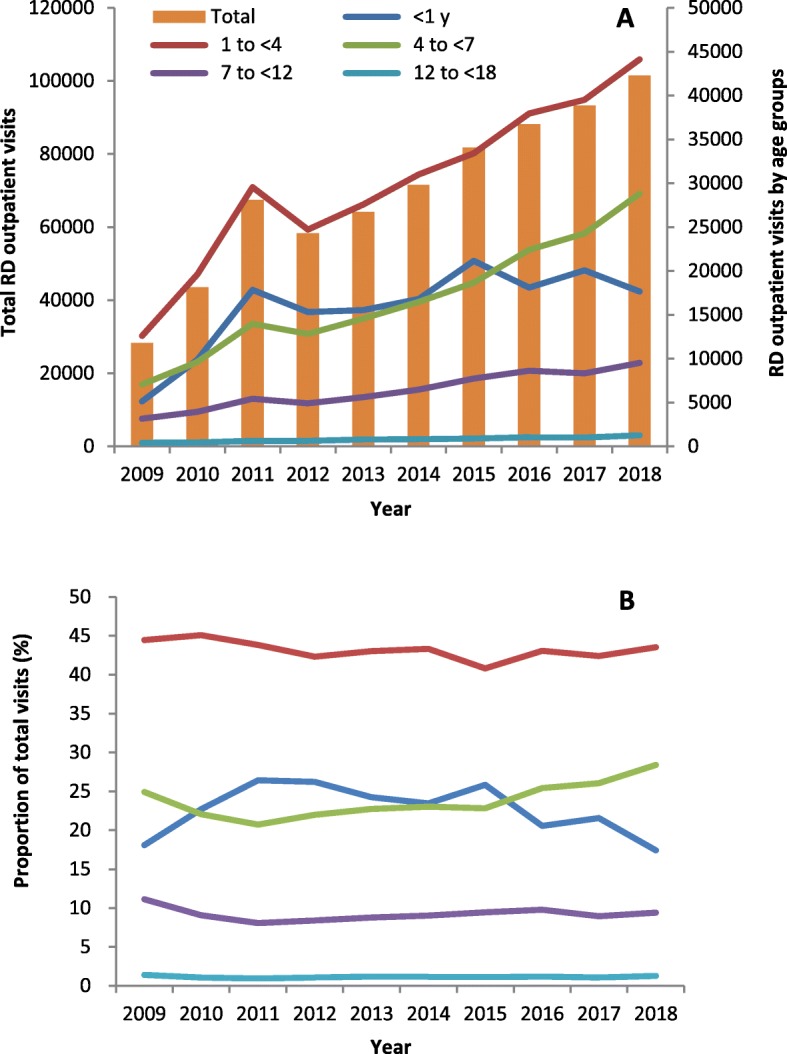
Time trends of visits and proportion of respiratory outpatient by age groups, 2009–2018

During the 10-year study period, the overall ROVs were 698,054, increasing from 28,329 visits in 2009 to 101,419 visits in 2018 with an AAGR of 15.2% (Fig. 2a).

### Trends of ROVs by age groups

Figure 2 displayed the time trends of total visits and visits by age groups (Fig. 2a), and the time trends of the proportions of different age groups accounting for total visits (Fig. 2b) during the study period. For children aged < 1 year, the annual visits increased over time with an AAGR of 14.7% (*P* < 0.001) and the proportion of total visits declined from 24.3% in 2013 to 17.4% in 2018 (*P* < 0.001). For children aged 1 to < 4 years, the AAGR were 14.9% with the stable 43% of total visits. For children aged 4 to < 7 years, the AAGR appeared the highest of 16.9% among all age groups (*P* < 0.001) and the proportion of total visits increased from 22.8% in 2013 to 28.4% in 2018 (*P* < 0.001). For children aged 7 to < 12 years and 12 to < 18 years, the AAGR increased slowly from 13.1 to 13.8% and they accounted for 9 and 1% of total visits, respectively.

### Trends of ROVs by gender

For male children, they accounted for 60.6% of total visits, from 62.9% in 2009 to 59.2% in 2018, with an AAGR of 14.4%. For female children, the proportion to total visits varied from 37.1% in 2009 to 40.8% in 2018 with an AAGR of 16.4%.

### Overall diagnoses distribution

**Fig. 3:**
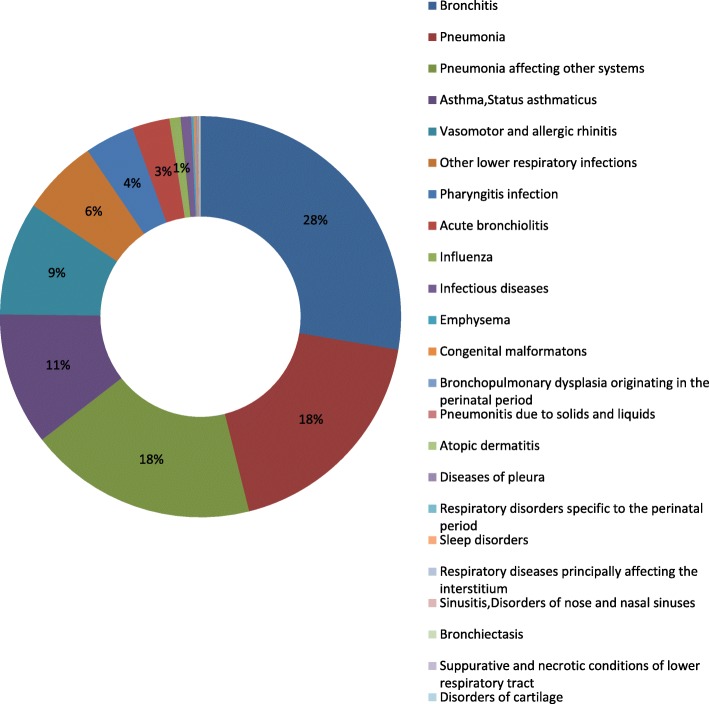
Distribution of diagnoses of respiratory diseases, 2009–2018

During the past 10 years, 23 different diagnoses categories were identified for all ROVs. Bronchitis was the most commonly identified diagnosis with 190,088 (27.6%) of total visits, followed by pneumonia (126,887, 18.5%), pneumonia affecting other systems (126,469, 18.4%), asthma and status asthmaticus (73,443, 10.7%), vasomotor and allergic rhinitis (62,951, 9.2%), other lower respiratory infections (42,353, 6.2%), and other respiratory diseases (65,261, 9.4%). The distributions of all the identified diagnoses were shown in Fig. 3.

### Gender- and age-specific trends in proportion of diagnoses

**Fig. 4:**
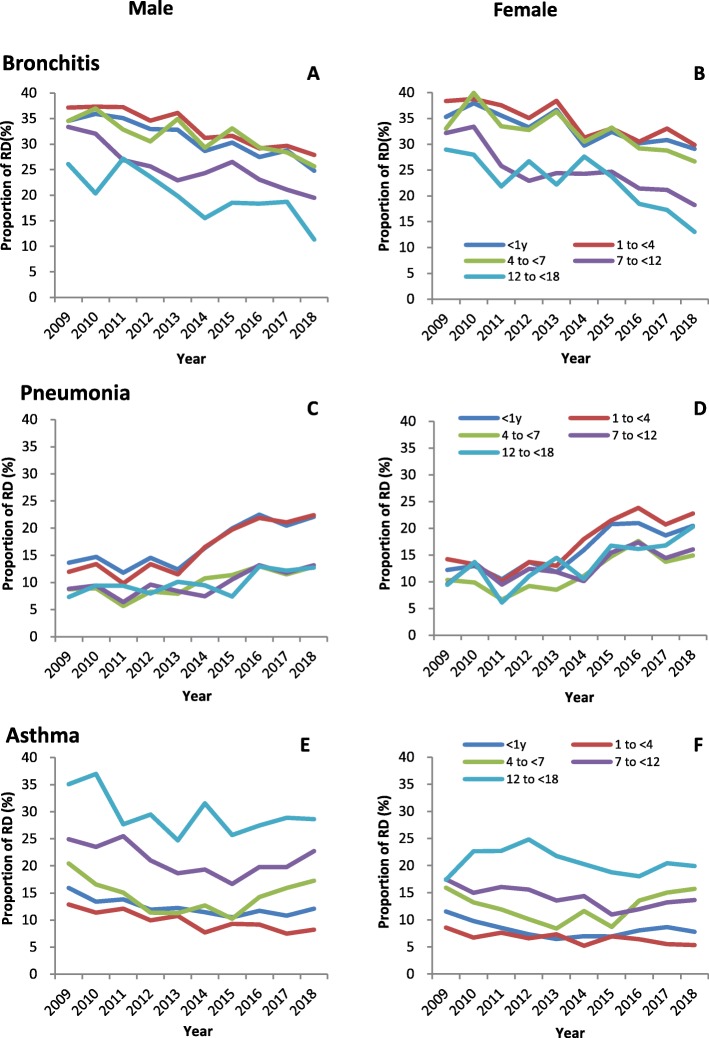
Time trends of proportion of respiratory diagnoses by gender and age groups, 2009–2018

Figure 4 showed the change of proportions of the selected three main diagnoses, bronchitis, pneumonia, asthma and status asthmaticus, stratified by gender and age groups.

For male children, the proportion of bronchitis in all ages declined from 34.8% in 2009 to 25.6% in 2018. In different age categories, the proportion of bronchitis for children aged < 1 year declined from 34.5 to 24.8%, aged 1 to < 4 years 37.1 to 27.9%, aged 4 to < 7 years 34.5 to 25.7%, aged 7 to < 12 years 33.4 to 19.5%, and aged 12 to < 18 years 26.1 to 11.3% (Fig. 4a). For female children, the proportion of bronchitis in all ages and age-specific categories showed the similar decline through the years (Fig. 4b).

For male children diagnosed pneumonia, the proportion of the disease in all ages increased from 13.0% in 2009 to 21.7% in 2018. Comparing the different age categories, children aged < 1 year and 1 to < 4 years accounted for larger proportion of pneumonia (13.6 and 11.96% in 2009; and 22.1 and 22.4% in 2018) than the older age categories, for example, male children aged 4 to < 7 years, 7 <  12 years, and 12 to < 18 years (4.8, 8.8, and 7.4% in 2009; and 12.9, 13.2, and 12.7% in 2018) (Fig. 4c). For female children, the proportion of pneumonia showed the similar increasing trend through the years (Fig. 4d).

For male children diagnosed asthma and status asthmaticus, the proportion of the diagnoses accounting for all visits decreased from 16.1% in 2009 to 10.3% in 2015, and then increased again to 11.5% in 2018. In age-stratified analysis for male children, the diagnoses (for example in 2009 and 2018) accounted for larger proportion in elder strata (35.1 and 28.6%, and 24.9 and 22.7% for 12 to < 18 years and 7 to < 12 years, respectively), while accounted for smaller proportion in younger strata (20.4 and 17.3%, 15.9 and 12.1%, and 12.9 and 8.2% for 4 to < 7 years, < 1 year, and 1 to < 4 years, respectively) (Fig. 4e). For female children, the proportion of asthma was lower than the male children at every age categories with similar change through the years. For example, 17.4% female children aged 7 to < 12 years were diagnosed asthma in 2009 and 13.6% in 2018, which were lower than those of the male children in the same age category (24.9% in 2009; 22.7% in 2018, respectively, *P* < 0.001) (Fig. 4f).

### Gender- and age-specific trends in visits of diagnoses

Table 2 showed the trends of the number of visits by respiratory diagnoses. For children diagnosed with bronchitis, the females had higher AAGR compared to the males in all age categories (14.6% vs. 10.39 for aged < 1 year, 12.9% vs. 10.8% for aged 1 to < 4 years, 15.5% vs. 12.4% for aged 4 to < 7 years, 7.4% vs. 6.0% for aged 7 to < 12 years, and 6.8% vs. 2.5% for aged 12 to < 18 year). Children aged < 7 years had higher AAGR than the older children for both the males and females (P for trend < 0.001).

**Table 2:** Gender- and age-specific trends in AAGR of visits by diagnosis, 2009–2018

	< 1 year		1 to < 4 years		4 to < 7 years		7 to < 12 years		12 to < 18 years	
	AAGR (%)	P for trend	AAGR (%)	P for trend	AAGR (%)	P for trend	AAGR (%)	P for trend	AAGR (%)	P for trend
Bronchitis										
Male	10.39	0.004	10.77	0.003	12.38	< 0.001	6.01	0.008	2.51	0.105
Female	14.62	< 0.001	12.90	0.001	15.49	< 0.001	7.37	0.002	6.75	0.066
Pneumonia										
Male	20.84	< 0.001	22.62	< 0.001	21.25	< 0.001	17.61	< 0.001	19.58	< 0.001
Female	23.98	< 0.001	22.30	< 0.001	23.17	< 0.001	21.06	< 0.001	27.03	< 0.001
Asthma and status asthmaticus										
Male	11.10	0.001	8.79	0.038	13.99	0.002	11.37	0.001	9.95	0.001
Female	12.12	< 0.001	10.11	0.009	18.06	0.001	11.24	< 0.001	18.43	0.001

For children diagnosed with pneumonia, they had nearly two fold AAGR compared to the children diagnosed with bronchitis and asthma. Female children had also higher AAGR than the males in all age categories, and the AAGR varied little between different age categories.

For children diagnosed with asthma and status asthmaticus, the AAGR was similar to that of the children with bronchitis, while it was higher than that of children with bronchitis aged > = 7 years. For each age category, female children with asthma had higher AAGR compared to the male children (P_s_ < 0.001).

## Discussion

Our study provided real world data of secular trends in outpatient visits and diagnosis amongst children visiting one of the largest children’s hospitals in China. Detailed computer records of this large sample sizes allowed investigating age- and gender-specific trends over a period of 10 years.

During the past 10 years, the trend in ROVs increased rapidly in the early years of the decade and slowed down substantially after 2013 (Fig. 2a). The result suggests a great increase in the demand of specialist medical service for respiratory diseases in the early years and nearly reaching the full level in the subsequent years.

Time trends of ROVs by age groups showed higher AAGR of the preschool children compared to the school children (aged 7 to < 18 years) (Fig. 2a). Among the total ROVs, preschool children (aged < 7 years) were the main consumers receiving specialist medical services. Children aged 1 to < 4 years accounted for 43.0% of ROVs in total, 4 to < 7 years accounted for a further 24.2%, and <  1 year accounted for 22.6%, respectively (Fig. 2b). This is understandable as preschoolers, with relatively naïve immune system, are the most susceptible to respiratory diseases and different environmental triggers. For gender trends in ROVs, boys were more susceptible to respiratory diseases compared to girls under the assumptions on stable population and sex ratio in the catchment areas of the hospital. This can be explained by that the sex-based DNA methylation signatures at birth and human respiratory conditions are largely influenced by sex resulting in overall higher risk for males than for females, particularly during early life [14].

When we further analyzed the trends in diagnoses of respiratory diseases, we found that bronchitis, pneumonia, asthma and status asthmaticus were main RODs, accounting for more than 80% of total visits during the past 10 years (Fig. 3). We explored the age- and gender-specific trends in number of visits and proportions of the selected diagnoses. The proportion of bronchitis decreased through the years (Fig. 4a, b), with a concomitant increasing trend in proportion of pneumonia (Fig. 4c, d). The disparity between ages indicates that preschool children had higher proportion of bronchitis or pneumonia compared to school children, and there were no statistically significant difference between males and females. Particularly when comparing the AAGRs of number of visits between males and females, we found consistently higher AAGRs of female children than of male children in all age categories (Table 2). Although males are more likely to be affected by infection-induced acute inflammation compared to females, the increasing trends in infection-induced respiratory disease burden for females should be aware of by pediatric physicians and policy makers [15].

For outpatient children diagnosed with asthma and status asthmaticus, we found a U-shape time trend of the proportion of asthma, which decreased till 2015 and then increased till 2018 (Fig. 4e, f). The variation of proportion of asthma remained in the range from 5 to 10% for all children (Fig. 4e, f). In China, guideline for the diagnosis and optimal management of asthma in children were first published in 2008 and then updated in 2016 [16, 17]. The publication of the criteria and awareness of asthma management influenced the variation of proportion of asthma in ROVs. The differential age- and gender-specific trends in proportion of asthma were observed in our study. The greater proportions of asthma were seen in children aged 12 to < 18 years and 7 to < 12 years compared with younger children, and consistently for both males and females (Fig. 4e, f). This finding was in accordance to the previous studies in the US, Europe, and China. The prevalence of asthma across all ages was 21.8% in Philadelphia, with an increased prevalence after 6–10 years of ages (22.9%) and to a peak between 14 and 17 years of age (23.0%) from 2001 to 2013 [18]. In Northern Sweden, the prevalence of physician-diagnosed asthma increased from 5.7% at age of 7–8 to 7.7% at age of 11–12 (*P* < 0.001) [19]. In Shanghai, China, the prevalence of childhood asthma showed greatly increasing from 1990 to 2011, and asthma was more reliably diagnosed in school children [10]. Asthma was in the process of increasing and the age of 4 to < 7 years is a critical period for pediatric physicians to take an early action on the treatment of asthma and allergic diseases. Considering the gender-specific trend in proportion and number of visits of asthma, our study showed the higher proportion of asthma in males than in females (Fig. 4e, f), while the greater AAGR among females (Table 2). The similar findings were also observed in the latest report in the US from 2001 to 2013. The US survey suggested asthma prevalence is higher among males in 5–9 years of age and increases among females compared to males in 10–17 years of age [9]. This gender-specific trend suggests the ‘adolescent switch’ discussed in other studies. Asthma mostly affects boys in childhood and women in adulthood [20], with many factors contributing to its frequency and severity, including shift in sex hormone [21–23], genetic factors [24], maternal asthma [25], and environmental exposure [26]. Gender-specific trend for asthma should be taken into account in asthma management and it is relevant to consider gender differences in the daily clinical practice [27].

### Strengths and limitations

One of the strengths of our study is the substantial large sample size of the 10-year administrative data. Second, we reported strictly in accordance to the RECORD statement. Last but not least, we used the ICD-10 classification system to analyze the age and gender-specific trend in RODs, which makes comparison with other studies possible.

There are also limitations in our study. Firstly, this is a hospital-based study in a single center. We cannot calculate the prevalence of pediatric RODs in the population. Therefore, we assumed the population and sex ratio stable in the catchment areas of the hospital (Additional file 1: Appendix 3). Comparing the proportion of respiratory diseases can be approximate to comparing the prevalence of the diseases. Secondly, the nature of retrospective study has a risk of report bias. The diagnosis recorded in the HIS system may have a higher occurrence of unspecified diagnosis and use of the ICD-10 category symptoms, signs and abnormal clinical and laboratory findings in the early of the decade. Thirdly, the bias derived from missing data in diagnosis cannot be excluded. We compared the distribution of age and gender of patients with missing data in diagnosis and no systematic difference was found (Additional file 1: Appendix 2). Lastly, our findings are based on data from a tertiary pediatric hospital, which may not be generalized to other suppliers of the pediatric medical service.

## Conclusion

Our findings suggest that the upward trend in pediatric ROVs among children at different ages in the past 10 years. More attention should be given to the children aged more than 4 years and between ‘adolescent switch’ who present an increasing trend of asthma. Gender differences and long-term prevention to control inflammation should be taken into account in daily clinical practice.

## Supplementary information

**Additional file 1 Appendix 1**: Diagnosis classification according ICD-10. **Appendix 2**: Missing rate and distribution comparison of gender and age. **Appendix 3**: Age and gender distribution of patients through 10 years, 2009–2018.

## Data Availability

The data collected and analyzed during the current study are available from the corresponding author on reasonable request.

## Abbreviations

AAGR: Average annual growth rate
HIS: Hospital Information System
ICD-10: The International Classification of Diseases, 10th Revision
IQR: Interquartile range
RD: Respiratory disease
RODs: Respiratory outpatient diagnoses
ROVs: Respiratory outpatient visits
