# Supplementary material for: Age- and gender-specific trends in respiratory outpatient visits and diagnoses at a tertiary pediatric hospital in China: a 10-year retrospective study
Source: BMC Pediatr. 2020 Mar 12;20:115. doi: 10.1186/s12887-020-2001-x (PMC7068978; doi:10.1186/s12887-020-2001-x)
Supplement: Supplementary file 1 — Additional file 1 Appendix 1: Diagnosis classification according ICD-10. Appendix 2: Missing rate and distribution comparison of gender and age. Appendix 3: Age and gender distribution of patients through 10 years, 2009–2018. [file 12887_2020_2001_MOESM1_ESM.docx]

**Appendix 1: Diagnosis classification according ICD-10**

| Chapter* | ICD-10 code | Disease name | Diagnosis categories | *n* of visits | % |
| --- | --- | --- | --- | --- | --- |
| I | A00-B99 | Certain infections and parasitic diseases | Infectious diseases | 5812 | 0.85 |
| II | C00-D48 | Neoplasms | Pneumonia ,AOS | 31 | 0.01 |
| III | D50-D89 | Diseases of the blood and blood-forming organs and certain disorders involving the immune mechanism | Pneumonia ,AOS | 243 | 0.04 |
| IV | E00-E90 | Endocrine, nutritional and metabolic diseases | Pneumonia ,AOS | 3469 | 0.50 |
| V | F00-F99 | Mental and behavioural disorders | Pneumonia ,AOS | 169 | 0.02 |
| VI | G00-G99 | Diseases of the nervous system | Pneumonia ,AOS | 149 | 0.02 |
|  |  |  | Sleep disorders (G47) | 216 | 0.03 |
| VII | H00-H59 | Diseases of the eye and adnexa | Other Lower respiratory infections, AOS | 81 | 0.01 |
| VIII | H60-H95 | Diseases of the ear and mastoid process | Other Lower respiratory infections, AOS | 67 | 0.01 |
| IX | I00-I99 | Diseases of the circulatory system | Pneumonia ,AOS | 104 | 0.02 |
| X | J00-J99 | Diseases of the respiratory system |  |  |  |
|  | J00-J06 | Acute upper respiratory infections | Pharyngitis infection | 27118 | 3.94 |
|  | J09-J18 | Influenza and pneumonia | Pneumonia | 126887 | 18.46 |
|  |  |  | Influenza (J10-J11) | 575 | 0.08 |
|  | J20-J22 | Other acute lower respiratory infections | Bronchitis (J20) | 15639 | 2.27 |
|  |  |  | Bronchiolitis (J21) | 20785 | 3.02 |
|  | J30-J39 | Other diseases of upper respiratory tract^*^ | Vasomotor and allergic rhinitis(J30-J31) | 62951 | 9.16 |
|  |  |  | Sinusitis, Disorders of nose and nasal sinuses(J32-J34) | 125 | 0.02 |
|  |  |  | Pharyngitis infection (J35-J38) | 221 | 0.03 |
|  |  |  | Influenza (J39) | 682 | 0.10 |
|  | J40-J47 | Chronic lower respiratory diseases^*^ | Bronchitis (J40,J42) | 173578 | 25.25 |
|  |  |  | Emphysema (J43,J44) | 1319 | 0.19 |
|  |  |  | Asthma, Status asthmaticus (J45,J46) | 73443 | 10.68 |
|  |  |  | Bronchiectasis (J47) | 125 | 0.02 |
|  | J60-J70 | Lung diseases due to external agents^*^ | Pneumonitis due to solids and liquids | 529 | 0.08 |
|  | J80-J84 | Other respiratory diseases principally affecting the interstitium | Respiratory diseases principally affecting the interstitium | 187 | 0.03 |
|  | J85-J86 | Suppurative and necrotic conditions of lower respiratory tract | Suppurative and necrotic conditions of lower respiratory tract | 60 | 0.01 |
|  | J90-J94 | Other diseases of pleura | Diseases of pleura | 291 | 0.04 |
|  | J95-J99 | Other diseases of the respiratory system | Pneumonia ,AOS | 122040 | 17.75 |
| XI | K00-K93 | Diseases of the digestive system | Other Lower respiratory infections, AOS | 1629 | 0.24 |
| XII | L00-L99 | Diseases of the skin and subcutaneous tissue | Atopic dermatitis | 352 | 0.05 |
| XIII | M00-M99 | Diseases of the musculoskeletal system and connective tissue | Pneumonia ,AOS | 79 | 0.01 |
|  |  |  | Disorders of cartilage (M94) | 27 | 0.01 |
| XIV | N00-N99 | Diseases of the genitourinary system | Pneumonia ,AOS | 148 | 0.02 |
| XV | O00-O99 | Pregnancy, childbirth and the puerperium | - | - | - |
| XVI | P00-P96 | Certain conditions originating in the perinatal period | Respiratory disorders specific to the perinatal period(P07) | 241 | 0.04 |
|  |  |  | Bronchopulmonary dysplasia originating in the perinatal period (P27) | 609 | 0.09 |
| XVII | Q00-Q99 | Congenital malformations, deformations and chromosomal abnormalities | Congenital malformations | 985 | 0.14 |
| XVIII | R00-R99 | Symptoms, signs and abnormal clinical and laboratory findings, not elsewhere classified | Other Lower respiratory infections, AOS (R05,R50) | 40576 | 5.90 |
|  |  |  | Bronchitis (R06) | 871 | 0.13 |
|  |  |  | Pharyngitis infection (R07) | 102 | 0.01 |
| XIX | S00-T98 | Injury, poisoning and certain other consequences of external causes | Pneumonia ,AOS | 37 | 0.01 |
| XX | V01-Y98 | External causes of morbidity and mortality | - | - | - |
| XXI | Z00-Z99 | Factors influencing health status and contact with health services | Influenza | 4900 | 0.71 |
| XXII | U00-U85 | Codes for special purposes | - | - | - |

*Source from: WHO, https://icd.who.int/browse10/2016/en

AOS：affecting other systems

**Appendix 2: Missing rate and distribution comparison of gender and age**

| Variables | 2009 | 2010 | 2011 | 2012 | 2013 | 2014 | 2015 | 2016 | 2017 | 2018 | Total | P value |
| --- | --- | --- | --- | --- | --- | --- | --- | --- | --- | --- | --- | --- |
| Total visits | 28329 | 43548 | 67457 | 58392 | 64115 | 71568 | 81851 | 88121 | 93254 | 101419 | 698054 |  |
| Diagnosis missing, visits | 543 | 778 | 949 | 855 | 1602 | 1718 | 1027 | 1235 | 1025 | 870 | 10602 |  |
| Missing rate of diagnosis (%) | 1.9 | 1.8 | 1.4 | 1.5 | 2.5 | 2.4 | 1.2 | 1.4 | 1.1 | 0.9 | 1.5 |  |
| Age, *n*(%) |  |  |  |  |  |  |  |  |  |  |  | <0.001 |
| <1 y | 132  (24.4) | 209  (26.8) | 272  (28.6) | 244  (28.5) | 379  (23.8) | 463  (27.0) | 305  (29.8) | 285  (23.2) | 280  (27.3) | 148  (17.0) | 2717  (25.7) |  |
| 1 to < 4 | 198  (36.3) | 305  (39.2) | 360  (37.9) | 347  (40.6) | 696  (43.4) | 745  (43.3) | 424  (41.3) | 531  (42.9) | 397  (38.7) | 395  (45.4) | 4398  (42.7) |  |
| 4 to < 7 | 124  (22.9) | 155  (20.0) | 213  (22.5) | 168  (19.7) | 366  (22.8) | 362  (21.1) | 202  (19.6) | 292  (23.6) | 253  (24.7) | 221  (25.4) | 2356  (22.0) |  |
| 7 to < 12 | 76  (14.0) | 92  (11.8) | 89  (9.4) | 78  (9.1) | 143  (8.9) | 130  (7.6) | 85  (8.2) | 111  (9.0) | 83  (8.1) | 85  (9.8) | 972  (8.4) |  |
| 12 to < 18 | 13  (2.4) | 17  (2.2) | 15  (1.6) | 18  (2.1) | 18  (1.1) | 18  (1.0) | 11  (1.1) | 16  (1.3) | 12  (1.2) | 21  (2.4) | 159  (1.2) |  |
| Sex, *n*(%) |  |  |  |  |  |  |  |  |  |  |  | 0.187 |
| Male | 345  (63.5) | 508  (65.3) | 587  (61.9) | 523  (61.2) | 977  (61.0) | 1077  (62.7) | 616  (60.0) | 737  (59.7) | 612  (59.7) | 517  (59.4) | 6499  (61.3) |  |
| Female | 198  (36.5) | 270  (34.7) | 362  (38.1) | 332  (38.8) | 625  (39.0) | 641  (37.3) | 411  (40.0) | 498  (40.3) | 413  (40.3) | 353  (40.6) | 4103  (38.7) |  |

**Appendix 3: Age and gender distribution of patients through 10 years, 2009-2018**

| Variables | 2009 | 2010 | 2011 | 2012 | 2013 | 2014 | 2015 | 2016 | 2017 | 2018 | Total | P value |
| --- | --- | --- | --- | --- | --- | --- | --- | --- | --- | --- | --- | --- |
| *n* of patients | 13837 | 18852 | 30712 | 27014 | 27257 | 27690 | 31128 | 32097 | 37233 | 39754 | 285574 |  |
| Payer type, *n*(%) |  |  |  |  |  |  |  |  |  |  |  | <0.001 |
| Government insurance | 7072  (51.1) | 8987  (47.7) | 13413  (43.7) | 11600  (42.9) | 11480  (42.1) | 11541  (41.7) | 12899  (41.4) | 14627  (45.6) | 17061  (45.8) | 18663  (46.9) | 127343  (44.6) |  |
| Self-finance | 6765  (48.9) | 9865  (52.3) | 17299  (56.3) | 15414  (57.1) | 15777  (57.9) | 16149  (58.3) | 18229  (58.3) | 17470  (54.4) | 20172  (54.2) | 21091  (53.1) | 158231  (55.4) |  |
| Sex, *n*(%) |  |  |  |  |  |  |  |  |  |  |  | <0.001 |
| Male | 8568  (61.9) | 11639  (61.7) | 18478  (60.2) | 16319  (60.4) | 16471  (60.4) | 16933  (61.2) | 18424  (59.2) | 18682  (58.2) | 21657  (58.2) | 23021  (57.9) | 170192  (59.6) |  |
| Female | 5269  (38.1) | 7213  (38.3) | 12234  (39.8) | 10695  (39.6) | 10786  (39.6) | 10757  (38.8) | 12704  (40.8) | 13415  (41.8) | 15576  (41.8) | 16733  (42.1) | 115382  (40.4) |  |
